# Causal relationship between fertility nutrients supplementation and PCOS risk: a Mendelian randomization study

**DOI:** 10.3389/fendo.2024.1420004

**Published:** 2024-09-24

**Authors:** Fang Shao, Shijia Xu, Haiyang Zhao, Furong Zhang, Xin Wang, Hui Wang

**Affiliations:** ^1^ Department of Biostatistics, School of Public Health, Nanjing Medical University, Nanjing, China; ^2^ Department of Histology and Embryology, School of Basic Medical Sciences, Nanjing Medical University, Nanjing, China; ^3^ Innovative Chinese Medicine Research Institute, Engineering Research Center of Modern Preparation Technology of Traditional Chinese Medicine, Shanghai University of Traditional Chinese Medicine, Shanghai, China; ^4^ State Key Laboratory of Reproductive Medicine and Offspring Health, Nanjing Medical University, Nanjing, China

**Keywords:** polycystic ovary syndrome, fertility nutrients, Mendelian Randomization, drug target Mendelian Randomization, reproductive health

## Abstract

**Background:**

Polycystic ovary syndrome (PCOS), a prevalent endocrine disorder in women of reproductive age, is mainly ameliorated through drugs or lifestyle changes, with limited treatment options. To date, numerous researchers have found that fertility nutrient supplements may benefit female reproductive health, but their direct impact on polycystic ovary syndrome risk remains unclear.

**Methods:**

Our research employs Mendelian Randomization to assess how fertility nutrients affect PCOS risk. Initially, we reviewed 49 nutrients and focused on 10: omega-3 fatty acids, calcium, dehydroepiandrosterone, vitamin D, betaine, D-Inositol, berberine, curcumin, epigallocatechin gallate, and metformin. Using methodologies of Inverse Variance Weighting and Mendelian Randomization-Egger regression, we examined their potential causal relationships with PCOS risk.

**Results:**

Our findings indicate omega-3 fatty acids reduced PCOS risk (OR=0.73, 95% CI: 0.57-0.94, *P*=0.016), whereas betaine increased it (OR=2.60, 95% CI: 1.09-6.17, *P*=0.031). No definitive causal relations were observed for calcium, dehydroepiandrosterone, vitamin D, D-Inositol, and metformin (*P*>0.05). Drug target Mendelian Randomization analysis suggested that increased expression of the berberine target gene BIRC5 in various tissues may raise PCOS risk (OR: 3.00-4.88; *P*: 0.014-0.018), while elevated expressions of curcumin target gene CBR1 in Stomach and epigallocatechin gallate target gene AHR in Adrenal Gland were associated with reduced PCOS risk (OR=0.48, P=0.048; OR=0.02, *P*=0.018, respectively).

**Conclusions:**

Our research reveals that specific fertility nutrients supplementation, such as omega-3 fatty acids, berberine, and curcumin, may reduce the risk of PCOS by improving metabolic and reproductive abnormalities associated with it.

## 1 Introduction

Polycystic Ovary Syndrome (PCOS) is a common metabolic disturbance and heterogeneous endocrine disorder among women of reproductive age (1), with an estimated prevalence of 6%-10% during this period (2, 3). It is mainly characterized by hyperandrogenism, anovulation, and/or oligo-ovulation. Studies have shown that women with PCOS exhibit persistent menstrual irregularities, infertility, and obesity. Moreover, even after conception, PCOS can lead to adverse pregnancy outcomes such as gestational hypertension, spontaneous miscarriages, and preterm births (4–6).

Despite its high prevalence, the exact etiology of PCOS remains unclear. Research indicates that PCOS is a multifactorial disease, often involving genetic, metabolic, and environmental abnormalities (7). Hyperandrogenism during different developmental stages is considered as a primary driver of the metabolic and reproductive dysregulations associated with PCOS (8). Insulin resistance is also commonly viewed as a significant etiological factor in PCOS, with approximately 50% to 80% of women with the syndrome exhibiting signs of insulin resistance (9, 10). Current treatment strategies for PCOS primarily involve lifestyle interventions or pharmacological treatments targeting pathological symptoms. Studies suggest that a weight reduction of 5% to 10% in overweight or obese women may restore regular menstruation and ovulation (11, 12); the use of metformin or D-chiro-D-Inositol can increase insulin sensitivity and improve the condition in patients with PCOS (13); and clomiphene citrate can block hypothalamic estrogen receptors, stimulating follicle development through a negative feedback mechanism (14). However, overall, the clinical drugs available are limited, and curing PCOS remains challenging.

Appropriate nutritional supplementation during preconception and pregnancy is beneficial for the development of the mother, embryos, fetuses, and pregnancy outcomes. These supplements are generally recognized as “Fertility Nutrients” or “Fertility Supplements” (15, 16). For example, supplementation with coenzyme Q10, melatonin, and vitamins A, C, and E can effectively promote follicular development and maturation in women with premature ovarian aging and diminished ovarian reserve (17–22). Supplementation with Docosahexaenoic acid (DHA), and minerals such as calcium, magnesium, and selenium, can effectively regulate hormone endocrine levels and insulin metabolism abnormalities in patients with PCOS (23–26). Supplementation with curcumin and vitamin D can effectively regulate overall blood glucose concentrations in pregnant women (21, 27). Additionally, supplementation with folic acid and betaine can significantly lower serum homocysteine levels and prevent fetal neural tube defects during preconception and pregnancy (18, 28). All of the above shows associations between nutrient supplementation and reproductive health, thus revealing whether reproductive nutrient supplementation can reduce the risk of PCOS is of great significance.

At present, common observational epidemiological research methods, such as meta-analyses and clinical randomized controlled trials, can associate medication or reproductive nutrient supplementation with PCOS. However, due to the difficulty in confirming whether all potential confounding factors and other possible influencing factors have been excluded from the correlation between exposure and outcome, the causal inference conclusions drawn from traditional analyses often lack rigor.

With the emergence of large-scale Genome-Wide Association Studies (GWAS) at the end of the 20th century, researchers have been able to use genetic variants to infer causal relationships between exposure factors (such as nutritional supplements, drugs, drug targets, or risk factors for disease) and outcome factors (such as diseases or biological events). Mendelian Randomization (MR) is a novel genetic analysis method that uses genetic variants strongly associated with exposure as instrumental variables to assess the causal relationship between exposure and outcome (29). This method relies on three essential assumptions: (1) Genetic variants are strongly associated with exposure. (2) Genetic variants are not associated with any confounders of the exposure-outcome relationship. (3) Genetic factors only affect the outcome through the exposure. MR avoids the confounding factors and reverse causality issues present in observational studies, providing a more reliable causal relationship than observational research.

Based on two-sample Mendelian Randomization studies, this research uses fertility supplements as the exposure factors and Polycystic Ovary Syndrome as the outcome to explore the causal relationship. This approach may provide more potential feasible solutions for the clinical treatment and prevention of PCOS.

## 2 Materials and methods

### 2.1 Acquisition and selection of exposure data

Using the IEU OpenGWAS database (https://gwas.mrcieu.ac.uk/) and the Drug Bank database (https://go.drugbank.com/), a search was conducted for 49 common fertility supplements (see **Supplementary Table 1**). A total of 10 supplements suitable for Mendelian Randomization (MR) analysis were selected for study, including DHA, calcium, Dehydroisoandrosterone (DHEA), vitamin D, betaine, and metformin, with their respective GWAS_IDs being met-d-DHA, ukb-b-7043, ebi-a-GCST004941, ebi-a-GCST90000618, met-a-362, and ukb-a-159 (**Table 1**). Data for D-Inositol (30) were downloaded from the GWAS Catalog (2023) website (https://www.ebi.ac.uk/gwas/).

**Table 1:** GWAS Data Information on Seven Fertility Nutritional Components.

GWAS MR			
Exposure	ID	N	Number of SNPs
DHA	met-d-DHA	114,999	12,321,875
Calcium	ukb-b-7043	461,384	9,851,867
DHEA	ebi-a-GCST004941	9,722	21,770,677
Vitamin D	ebi-a-GCST90000618	496,946	6,896,093
Betaine	met-a-362	7,806	2,545,684
Metformin	ukb-a-159	337,159	10,894,596
Inositol	/	8193	/

Exposure, Exposure factor; ID, GWAS_ID; N, Sample size of GWAS data; Number of SNPs, Number of SNPs detected in GWAS data samples.

Additionally, berberine, curcumin, and epigallocatechin gallate (EGCG) were searched, and 10 target genes were identified respectively as BIRC5, qacR, PPARG, VDR, ABCC5, CBR1, GSTP1, AHR, DNMT1, and DHFRL1 (**Table 2**). Public data from GTEx (https://www.gtexportal.org/) were utilized to obtain the cis-eQTLs associated with the expression of these target genes in 49 human tissues. These cis-eQTLs were used as instrumental variables for drug target MR analysis.

**Table 2:** Fertility nutrients target data information.

Nutrient composition Target MR		
Exposure	Target	Number of SNPs
Berberine	BIRC5	389
	qacR	0
Curcumin	PPARG	1,058
	VDR	530
	ABCC5	4,150
	CBR1	4,809
	GSTP1	5,693
EGCG	AHR	88
	DNMT1	76
	DHFRL1	0

Exposure, Exposure Factor; Target, Drug Target; Number of SNPs, Number of SNPs Corresponding to Targets. BIRC5, Baculoviral IAP repeat-containing protein 5; qacR, HTH-type transcriptional regulator QacR; PPARG, Peroxisome proliferator-activated receptor gamma; VDR, Vitamin D3 receptor; ABCC5, Multidrug resistance-associated protein 5; CBR1, Carbonyl reductase [NADPH] 1; GSTP1,Glutathione S-transferase P; AHR, Aryl hydrocarbon receptor; DNMT1, DNA (cytosine-5)-methyltransferase 1; and DHFRL1, Dihydrofolate reductase, mitochondrial.

### 2.2 Acquisition of outcome data

The GWAS_ID for PCOS was retrieved from the IEU OpenGWAS project database (https://gwas.mrcieu.ac.uk/) as finn-b-E4_POCS. This data originates from publicly published GWAS summary statistics in 2021, encompassing a total of 16,379,676 SNPs.

### 2.3 Two-sample MR analysis

We conducted a two-sample Mendelian Randomization (MR) analysis (31) using R software (version 4.2.3) and the TwoSampleMR package (version 0.5.7). For the seven fertility nutritional components with GWAS_ID, causal relationships with PCOS were assessed using mendelian randomization analysis methods (instrumental variable logistic regression with SNPs as instrumental variables) such as inverse variance weighted (IVW), MR-Egger regression, simple mode, weighted median, and weighted mode. Odds ratios (OR) and their 95% confidence intervals (CI) were estimated, and a *p*-value < 0.05 was considered statistically significant.

Heterogeneity was tested using Cochran’s Q test, with a *p*-value < 0.05 indicating the presence of heterogeneity. Pleiotropy was assessed using the MR-Egger intercept test, with a *p*-value < 0.05 indicating the presence of pleiotropy. Sensitivity analysis was conducted using the leave-one-out method to evaluate the robustness of the findings, checking if the removal of a single variant affected the relationship between exposure and outcome. Forest plots were used to visualize causal associations, and scatter plots were used to visualize the effect sizes of each genetic instrument on exposures and outcomes.

### 2.4 Drug target MR analysis

For the three fertility nutritional components without GWAS data, we analyzed the instrumental variables containing multiple SNP loci using a fixed-effect inverse variance weighted MR method. For instrumental variables containing only a single SNP locus, the Wald ratio method was used.

## 3 Results

**Figure 1:**
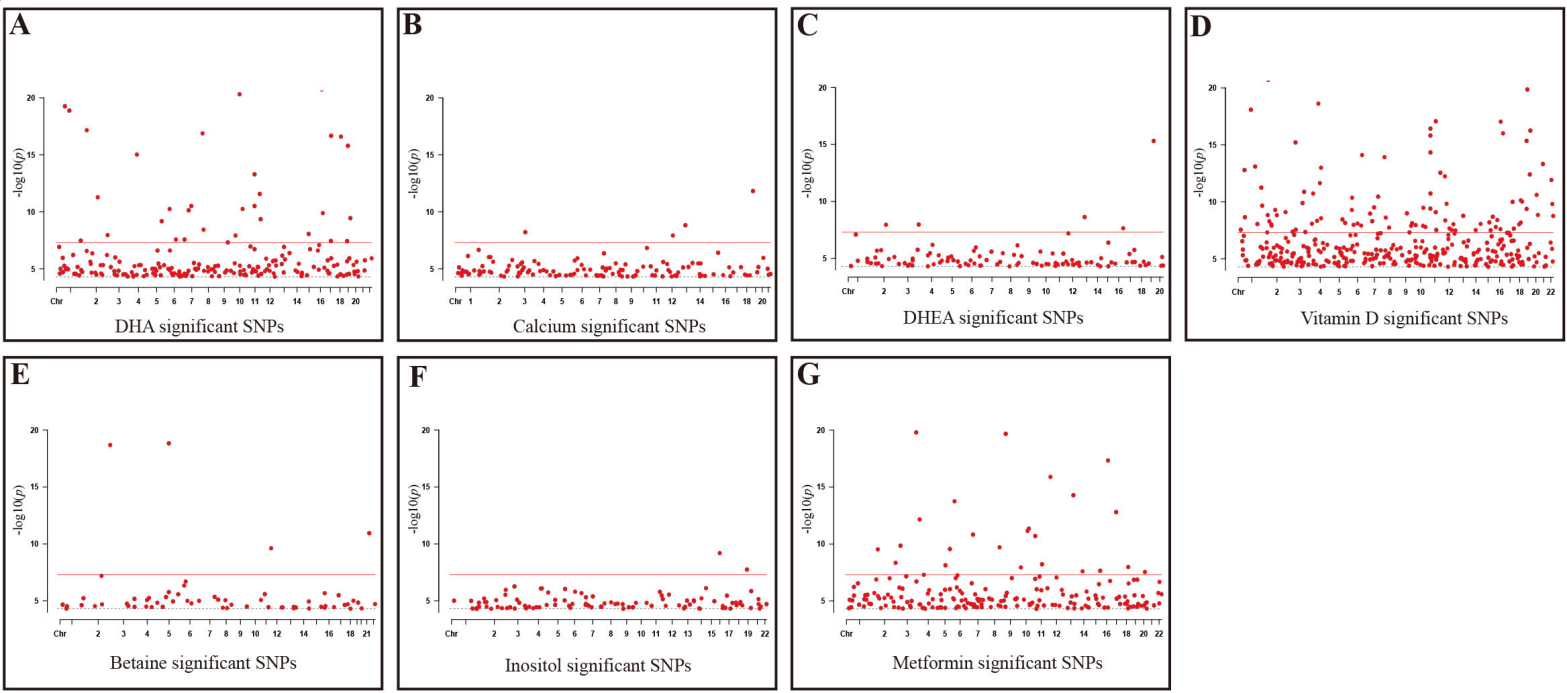
Significant SNPs in GWAS Data for reproductive nutritional components. **(A)** DHA; **(B)** Calcium; **(C)** DHEA; **(D)** Vitamin D; **(E)** Betaine; **(F)** Inositol; **(G)** Metformin. X-axis: Chromosomal positions of SNPs; Y-axis: SNPs correspond to negative log10 values of *p*-values; Gray dashed line: *P*=5e-5 threshold; Red solid line: *P*=5e-8 threshold.

Firstly, this study took seven reproductive nutritional supplements with GWAS data—DHA, calcium, DHEA, vitamin D, betaine, D-Inositol, and metformin—as exposure factors (**Table 1**). After genome-wide screening (*p* < 5 × 10^-5) and clumping (r^2 < 0.001, kb = 10,000), 216, 126, 104, 376, 61, 99, and 196 SNPs were included respectively. These SNPs were strongly associated with the exposure factors and were independent of each other (**Figure 1**).

**Figure 2:**
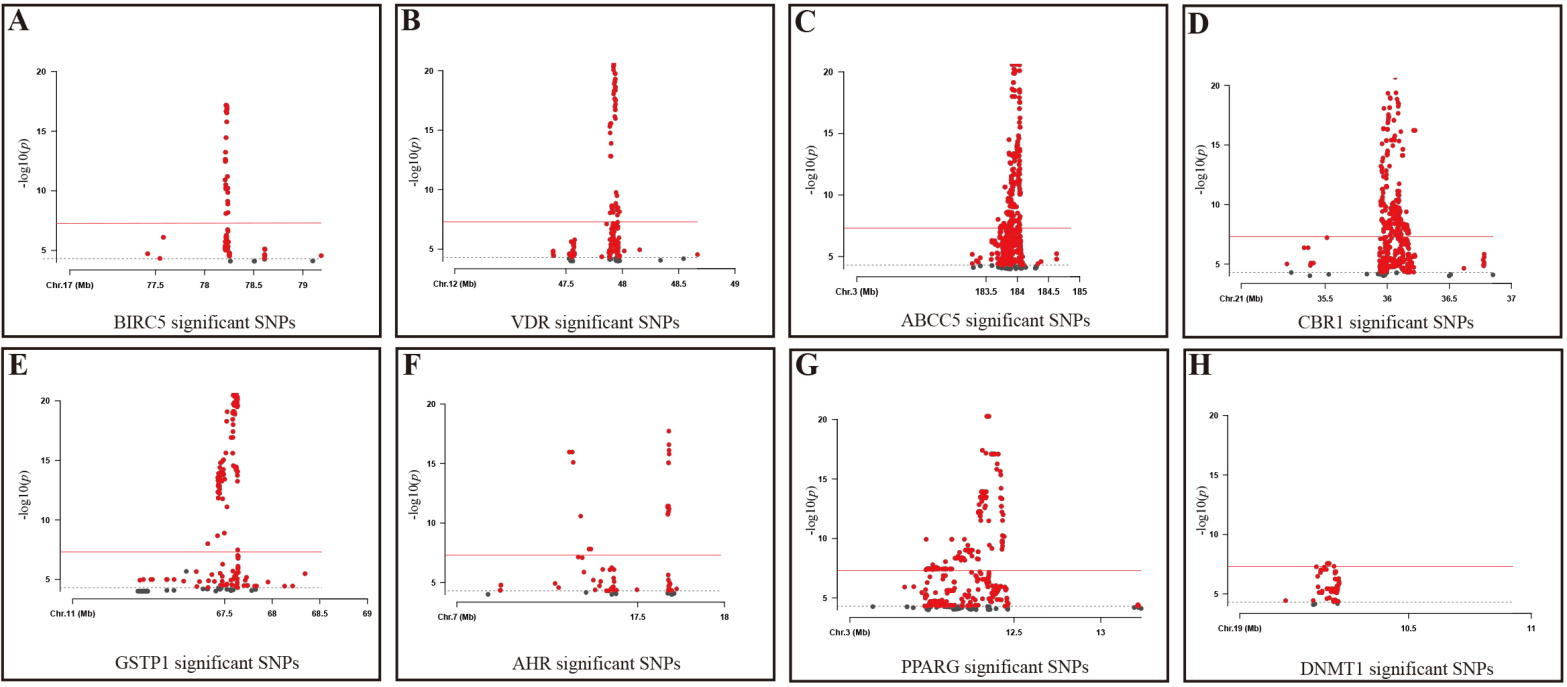
Significant SNPs in Target Genes for fertility nutritional components. **(A)** BIRC5; **(B)** VDR; **(C)** ABCC5; **(D)** CBR1; **(E)** GSTP1; **(F)** AHR; **(G)** PPARG; **(H)** DNMT1; Gray dashed line: *P*=5e-5 threshold; Red solid line: *P*=5e-8 threshold. X-axis: Chromosomal position of the SNP; Y-axis: SNPs correspond to negative log10 values of *p*-values.

At the same time, this study used the eQTL data corresponding to the target action points of three fertility nutritional components without GWAS data—berberine, curcumin, and EGCG—as instrumental variables. After screening (*p* < 5 × 10^-5), the target genes for berberine: BIRC5 and qacR, included 389 and 0 SNPs respectively; the target genes for curcumin: PPARG, VDR, ABCC5, CBR1, and GSTP1, included 1058, 530, 4150, 4809, and 5693 SNPs respectively; the target genes for EGCG: AHR, DNMT1, and DHFRL1, included 88, 76, and 0 SNPs respectively (**Figure 2**).

**Figure 3:**
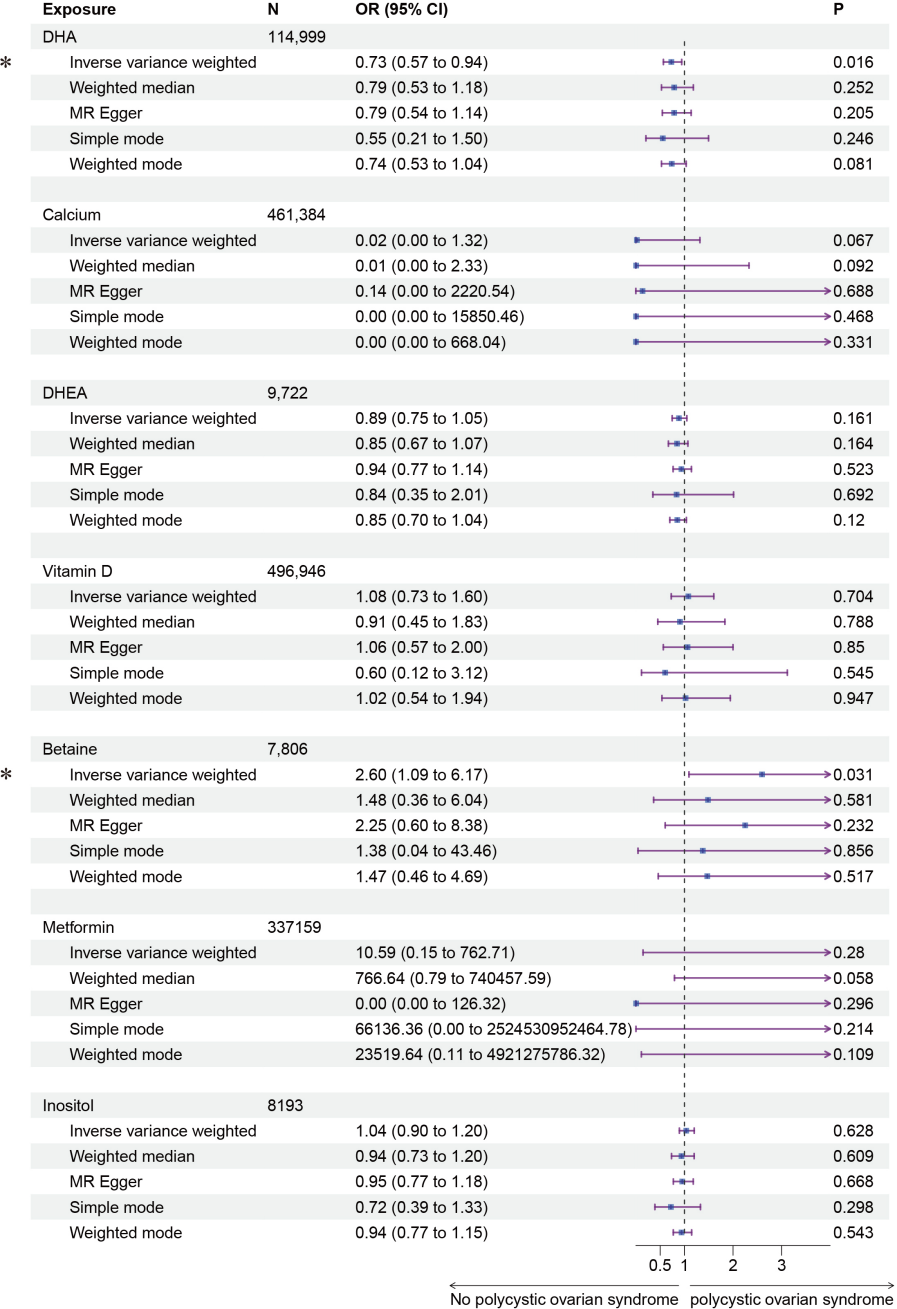
Forest plot of the results of MR Analysis of fertility nutrients and PCOS. Exposure, Exposure Factor; N, Sample Size; OR, Odds Ratio; 95% CI, 95% Confidence Interval; *P*, *p*-value; an asterisk “*” denotes *P*<0.05 as a statistically significant result in the Mendelian Randomization analysis.

**Figure 4:**
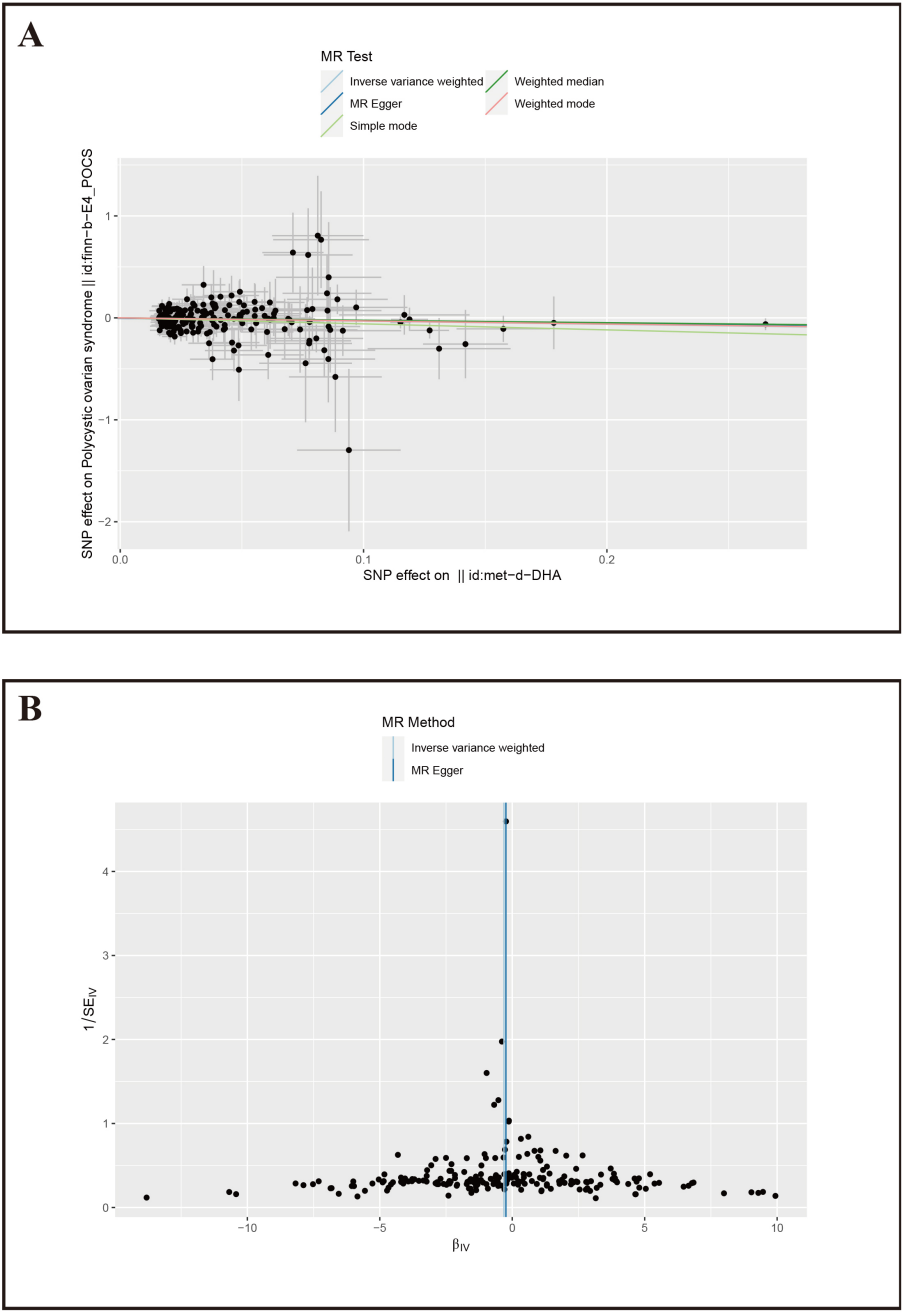
Mendelian Randomization results for DHA and PCOS. **(A)** Scatter plot of SNP effects on PCOS, X-axis: Effect values of SNPs on DHA; Y-axis: Effect values of SNPs on PCOS. **(B)** Volcano plot, βIV: Instrumental variable effect value; 1/SE_IV_: Inverse of the standard error.

Subsequently, we conducted a two-sample MR analysis on the seven fertility nutritional supplements with GWAS data. The IVW results showed that DHA could decrease the risk of PCOS (OR=0.73, 95% CI: 0.57-0.94, *P*=0.016), while betaine could increase the risk of PCOS (OR=2.60, 95% CI: 1.09-6.17, *P*=0.031). For calcium, DHEA, vitamin D, D-Inositol, and metformin, the MR analyses across all five regression models showed *p*-values greater than 0.05, indicating no statistical significance (**Figure 3**). In MR analysis, the IVW regression analysis is of particular importance; if the IVW result is statistically significant, and the effect value (b) of other non-statistically significant models is in the same direction as the IVW b value, the exposure factor can still be considered to have a causal relationship with the outcome. The scatter plot for DHA and PCOS is shown in **Figure 4A**. The forest plot for the effect estimates of individual SNPs on PCOS is provided in **Supplementary Figure 1**.

Furthermore, we conducted sensitivity tests for the MR analysis results of DHA and PCOS. The IVW results showed Cochran’s Q=203.16, *P*=0.50; MR-Egger results showed Cochran’s Q=203.46, *P*=0.52, with both methods having *p*-values greater than 0.05, indicating no heterogeneity among the instrumental variables. The MR-Egger intercept was 0.0045, *P*=0.58, suggesting no pleiotropy among the included SNPs. The funnel plot also indicated that the causal effect estimates of IVW were symmetrically distributed overall, showing no heterogeneity (**Figure 4B**). The study also used the Leave-one-out method to test the impact of the remaining SNPs after removing a single SNP, and it was observed that there were no outlier SNPs or SNPs affecting the results (**Supplementary Figure 2**), thereby confirming the reliability of our results.

**Figure 5:**
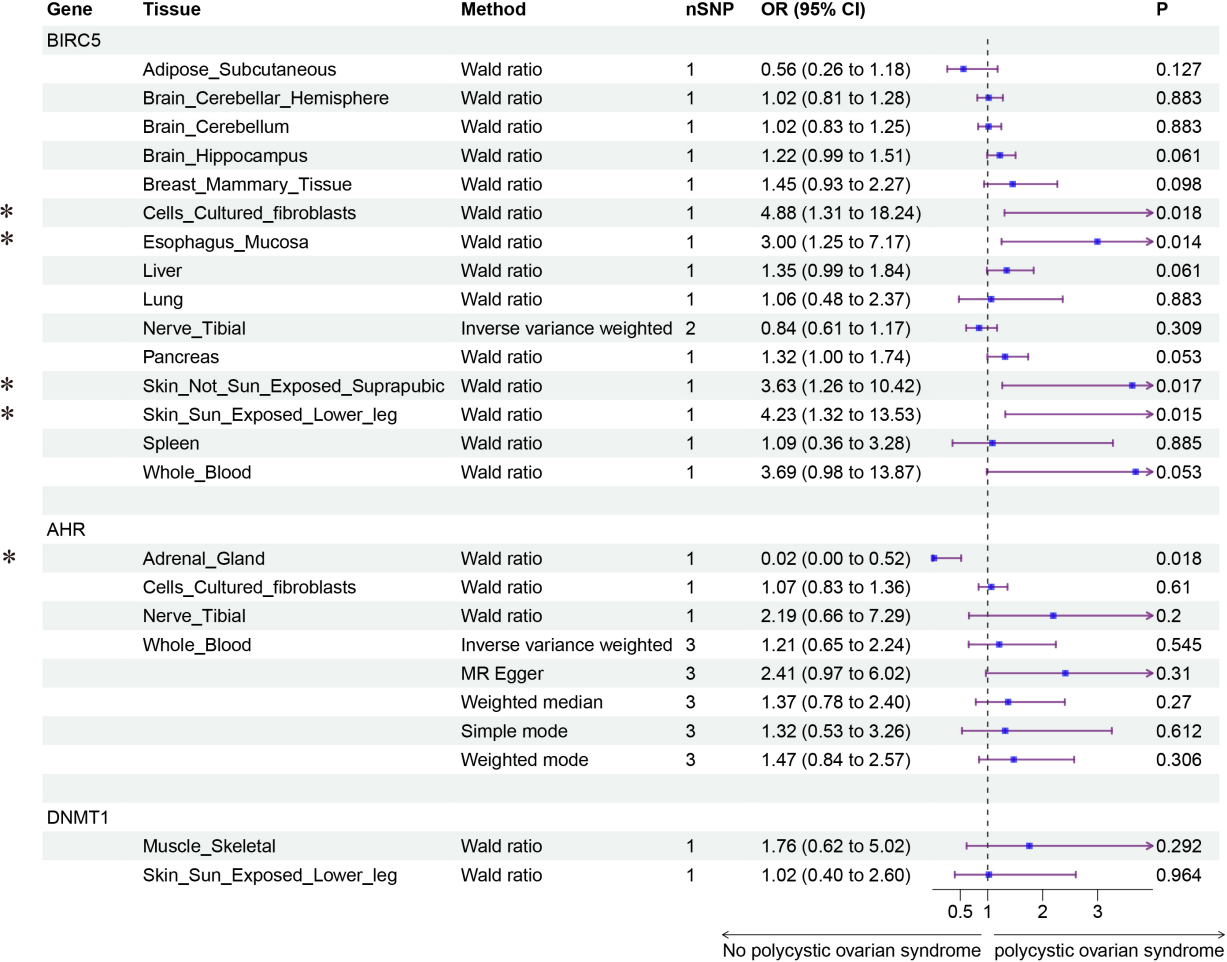
Forest plot of the results of MR Analysis of berberine (BIRC5) and EGCG (AHR and DNMT1) target gene SNPs and PCOS. Gene, Target gene; Tissue, Tissue where SNPs are located; nSNPs, Number of effective SNPs (*P*<5e-5); OR, Odds Ratio; 95% CI, 95% Confidence Interval; P, *p*-value corresponding to SNPs in MR results; an asterisk “*” denotes *P*<0.05 as a statistically significant result in the Mendelian Randomization analysis.

**Figure 6:**
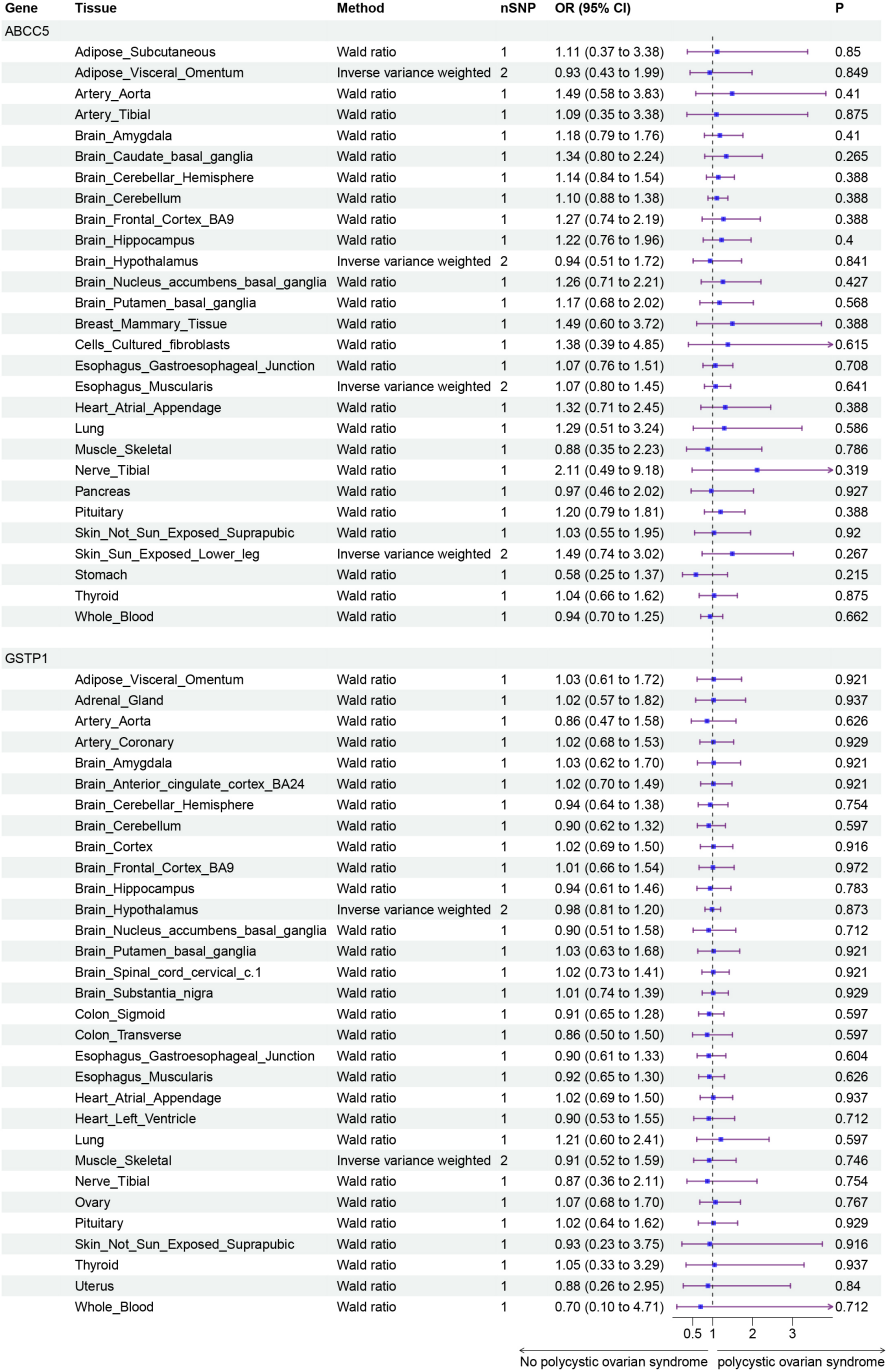
Forest plot of the results of MR Analysis of curcumin target gene (ABCC5 and GSTP1) SNPs and PCOS. Gene, Target gene; Tissue, Tissue where SNPs are located; nSNPs, Number of effective SNPs (*P*<5e-5); OR, Odds Ratio; 95% CI, 95% Confidence Interval; P, *p*-value corresponding to SNPs in MR results.

**Figure 7:**
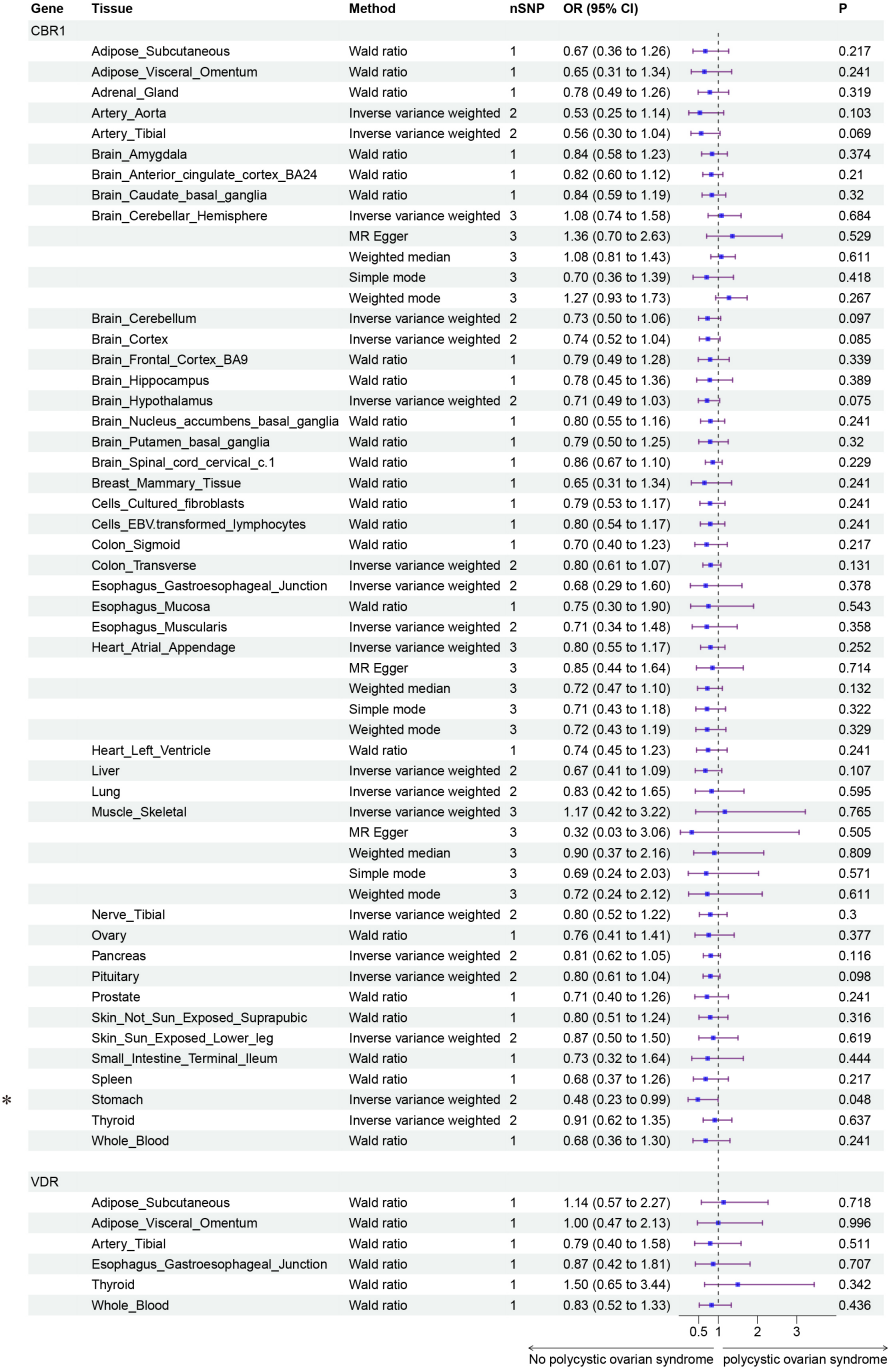
Forest plot of the results of MR Analysis of curcumin (CBR1 and VDR) target gene SNPs and PCOS. Gene, Target gene; Tissue, Tissue where SNPs are located; nSNPs, Number of effective SNPs (*P*<5e-5); OR, Odds Ratio; 95% CI, 95% Confidence Interval; P, *p*-value corresponding to SNPs in MR results; an asterisk “*” denotes *P*<0.05 as a statistically significant result in the Mendelian Randomization analysis.

Finally, we conducted Mendelian randomization (MR) analyses on the drug targets of three fertility nutritional components: berberine, curcumin, and EGCG. The results indicated that the upregulation of the berberine target gene BIRC5 in tissues such as “Cells _ Cultured _ fibroblasts”, “Esophagus _ Mucosa”, “Skin _ Not _ Sun _ Exposed _ Suprapubic”, and “Skin _ Sun _ Exposed _ Lower _ leg” could increase the risk of PCOS (OR=4.88, 95% CI: 1.31-18.24, *P*=0.018; OR=3.00, 95% CI: 1.25-7.17, *P*=0.014; OR=3.63, 95% CI: 1.26-10.42, *P*=0.017; OR=4.23, 95% CI: 1.32-13.53, *P*=0.015 respectively). In Adrenal _ Gland tissue, the upregulation of the EGCG target gene AHR could decrease the risk of PCOS (OR=0.02, 95% CI: 0.00-0.52, *P*=0.018), and in Stomach tissue, the upregulation of the curcumin target gene CBR1 could decrease the risk of PCOS (OR=0.48, 95% CI: 0.23-0.99, *P*=0.048) (**Figures 5**–**7**).

## 4 Discussion

Polycystic Ovary Syndrome (PCOS) is a common reproductive endocrine metabolic disease among women of reproductive age, characterized by hyperandrogenism, ovulatory dysfunction, and the presence of polycystic ovaries (32). Due to the heterogeneity of these features, diagnosing and treating PCOS has always been challenging. This study utilized a two-sample Mendelian randomization approach to assess the causal relationship between reproductive nutritional supplementation and the risk of developing PCOS.

In this study, we evaluated the potential causal relationship between fertility nutritional components, using GWAS data, and the risk of PCOS. The components assessed included DHA, calcium, DHEA, vitamin D, betaine, D-Inositol, metformin. The results suggest that DHA may reduce the risk of PCOS (OR=0.73, 95% CI: 0.57-0.94, *P*=0.016), while betaine may increase the risk of PCOS (OR=2.60, 95% CI: 1.09-6.17, *P*=0.031). No significant associations were found between the other nutritional components and PCOS.

Women with PCOS often exhibit insulin resistance, characterized by excessive insulin secretion and reduced glucose utilization by insulin (33). Numerous basic and clinical studies have shown that DHA (omega-3 polyunsaturated fatty acids) can beneficially impact PCOS through various pathways and mechanisms, including reducing insulin resistance, modulating adipokine production, exerting anti-inflammatory effects, and enhancing endothelial function (34–36). Consistent with these findings, our study also indicates that DHA intake decreases the risk of PCOS.

Metformin, as an insulin sensitizer, is widely used to ameliorate insulin resistance in patients with PCOS. However, the reduction in gastrointestinal glucose absorption caused by metformin can lead to symptoms such as nausea, vomiting, and diarrhea, which may interrupt PCOS treatment (33). Hence, alternatives with fewer side effects would be beneficial for the recovery of PCOS patients. D-Inositol has long been considered a potential substitute for metformin in the treatment of PCOS. A meta-analysis has indicated that D-Inositol supplementation for PCOS can help normalize the ovarian cycle, regulate carbohydrate metabolism, and significantly improve hyperandrogenemia symptoms by reducing free and total testosterone levels and increasing sex hormone-binding globulin levels (37). However, our study’s findings show that increased levels of metformin and D-Inositol do not have a significant causal relationship with the risk of PCOS, indicating that the currently reported GWAS data related to those compounds are limited. Therefore, MR serves as a predictive tool, and its results have suggestive value, and further observational studies and clinical trials are warranted.

During the treatment of patients with PCOS, in addition to improving insulin resistance, reducing homocysteine levels may also enhance reproductive outcomes (38, 39). As a methyl donor, betaine works in conjunction with betaine-homocysteine methyltransferase to convert homocysteine into methionine, thereby lowering homocysteine levels (40). This suggests that betaine supplementation could improve symptoms of hyperhomocysteinemia in PCOS patients, thereby improving PCOS outcomes. However, our MR study results indicated that increased levels of betaine might exacerbate the risk of PCOS, which contradicts previous research. The causal relationship between betaine levels and PCOS lacks direct clinical evidence and requires further investigation to explore this relationship.

For the fertility nutritional components without GWAS data—berberine, curcumin, and EGCG—we conducted the analysis using drug target genes as instrumental variables. Our findings suggest that the elevated expression of the target gene BIRC5 for berberine is linked to a heightened risk of PCOS, while increased expression of target genes for curcumin and EGCG is associated with a reduced risk of PCOS.

Berberine is an alkaloid with a variety of pharmacological effects, including anti-inflammatory, antioxidant, and insulin sensitivity-improving actions (41, 42). This study identified that the elevated expression of the berberine target gene BIRC5 across various tissues is associated with an increased risk of PCOS. The protein encoded by BIRC5, SURVIVIN, is a member of the cell cycle regulatory proteins involved in inhibiting apoptosis and promoting cell proliferation (43). Elevated expression of BIRC5 is associated with the development of various cancers, and abnormal expression of BIRC5 has also been found in the ovarian granulosa cells of PCOS patients (44, 45). Hao et al. have indicated that alkaloids like berberine and cantharidin may reduce the levels of proteins such as BIRC5, CDK1, and CCNB1 to combat chronic hepatitis B virus (46). Thus, berberine might exert its therapeutic effects on PCOS by suppressing the expression of the BIRC5 gene, thereby decreasing the expression of SURVIVIN, ultimately improving PCOS outcomes. However, this hypothesis requires further validation through more cellular and animal studies.

Curcumin, the principal active component of turmeric, is known for its extensive anti-inflammatory and antioxidant properties and has been shown to play a role in the management of diabetes and cardiovascular diseases (47, 48). In our study, the expression of the curcumin target gene CBR1 in stomach tissue was associated with a reduced risk of PCOS. CBR1 is a reductase involved in drug metabolism and cellular antioxidative defense mechanisms (49). The regulatory effect of curcumin on CBR1 may be linked to its potential in ameliorating metabolic abnormalities in PCOS patients, but the specific mechanisms and efficacy of this action remain to be further explored.

Epigallocatechin gallate (EGCG), one of the most active components in green tea, exhibits significant antioxidant, anti-inflammatory, and potential anticancer effects (50, 51). In this study, the expression of the target gene AHR for EGCG in adrenal tissue was significantly associated with a reduced risk of PCOS. However, studies have shown that Bisphenol A induces insulin resistance, a potential factor in the development of PCOS, by activating the AHR receptor to inhibit GLUT4 expression, leading to aberrant glucose metabolism (52). This finding is inconsistent with our MR results, and the specific mechanism by which AHR affects PCOS requires further research.

The aforementioned findings provide potential directions for both clinical and basic research into the treatment and prevention of PCOS. These nutritional components may improve clinical outcomes in PCOS by affecting the expression of specific genes and related biological pathways.

Mendelian randomization analysis is a powerful tool for studying causal relationships, addressing confounding and reverse causality issues in traditional observational studies by using genetic variants as instrumental variables. In our study, we employed multiple MR methods to test causal relationships and conducted heterogeneity and pleiotropy tests, as well as sensitivity analyses, to ensure the robustness of our results. Despite the enhanced credibility of our analyses through these methods, our study still has some limitations.

Firstly, our analysis relies on existing GWAS datasets, which may lack diversity in terms of ethnicity, geography, and environmental background, thereby potentially limiting the generalizability of our findings. For example, the PCOS GWAS data utilized in our study are derived solely from European populations, rather than global populations. Additionally, due to the limitations of GWAS summary data, Mendelian randomization analysis can only estimate odds ratios (OR), even though relative risk (RR) is more appropriate for estimating risk.

Secondly, the association between the nutrients we studied and PCOS might be influenced by other genetic and environmental factors that were not fully controlled for in our analysis. For example, calcium’s effect approached borderline significance (*P* = 0.067) and was favorable in reducing PCOS. The larger standard error and wider confidence interval could be due to the sample size, suggesting calcium as a potential beneficial factor warranting further investigation. Our results may be limited by the strength of the association between the genetic variants used as instrumental variables and the exposure to nutritional components, as well as the number of these genetic variants.

Nevertheless, our study provides new insights, identifies potential genetic variants, and offfers targeted research recommendations for further clinical research and the development of therapeutic strategies.

## 5 Conclusions

In conclusion, our research suggests that specific fertility nutritional components, such as DHA, berberine, and curcumin, may reduce the risk of PCOS by improving metabolic and reproductive abnormalities. Future research should focus on verifying the therapeutic efficacy and mechanisms of action of these nutrients on PCOS, while also assessing their potential values in the prevention and treatment of PCOS. Attention should also be given to the relationship between nutrient supplementation and other phenotypes of PCOS. Furthermore, direct intervention studies, such as randomized controlled trials, can better elucidate the impact of nutritional supplementation strategies on PCOS and provide more specific guidance for patients. It is important to note that our current data are derived from European cohorts and may not be broadly representative. The applicability of our conclusions to populations outside Europe requires further validation and analysis.

## Data Availability

The original contributions presented in the study are included in the article/**Supplementary Material**. Further inquiries can be directed to the corresponding authors.
