# Supplementary material for: Causal relationship between fertility nutrients supplementation and PCOS risk: a Mendelian randomization study
Source: Front Endocrinol (Lausanne). 2024 Sep 24;15:1420004. doi: 10.3389/fendo.2024.1420004 (PMC11458446; doi:10.3389/fendo.2024.1420004)

**Supplementary Table 1. Fertility Supplement Screening Catalogue**

| Fertility nutritional supplementation | | | | | | |
| --- | --- | --- | --- | --- | --- | --- |
| Panthenol | Vitamin E | Carob bean extract | Berberine | Black cohosh | Biotin | Ergothionein |
| Ubiquinone | Nattokinase | DHA | Metformin | Soy isoflavones | Parsley | Glutathione |
| Resveratrol | Iron | EPA | Curcumin | Puerarin | South African drunk nightshade | DIM |
| DHEA | Acetyl L-carnitine | Calcium | Melatonin | Saw palm extract | Astaxanthin | I3C |
| Active folic acid | L-arginine | Icariin | Taurine acid | Quercetin | Alpha lipoic acid | Extract of vitex |
| Vitamin D3 | Zinc | Lycopene | Dodder seed | EGCG | Sulforaphane | Whey protein |
| NAC | Selenium | Oyster oligopeptide | Betaine | Choline | Bromelain | Inositol/  D-Inositol |

**Supplementary Table 2. Abbreviation comparison table**

| Full term | Abbreviation |
| --- | --- |
| Polycystic ovary syndrome | PCOS |
| Odds ratios | OR |
| Docosahexaenoic acid | DHA |
| Genome-Wide Association Studies | GWAS |
| Mendelian Randomization | MR |
| Dehydroisoandrosterone | DHEA |
| Epigallocatechin gallate | EGCG |
| Baculoviral IAP repeat-containing protein 5 | BIRC5 |
| HTH-type transcriptional regulator QacR | qacR |
| Peroxisome proliferator-activated receptor gamma | PPARG |
| Vitamin D3 receptor | VDR |
| Multidrug resistance-associated protein 5 | ABCC5 |
| Carbonyl reductase [NADPH] 1 | CBR1 |
| Glutathione S-transferase P | GSTP1 |
| Aryl hydrocarbon receptor | AHR |
| DNA (cytosine-5)-methyltransferase 1 | DNMT1 |
| Dihydrofolate reductase, mitochondrial | DHFRL1 |
| Inverse variance weighted | IVW |
| Confidence intervals | CI |
| Relative risk | RR |
| Cyclin-dependent kinase 1 | CDK1 |
| Cyclin B1 | CCNB1 |
| Glucose transporter type 4 | GLUT4 |
| Eicosapentaenoic Acid | EPA |
| 3,3'-Diindolylmethane | DIM |
| Indole-3-carbinol | I3C |
| N-Acetylcysteine | NAC |

**Supplementary Figure 1. Forest plot of MR Results for DHA and PCOS**


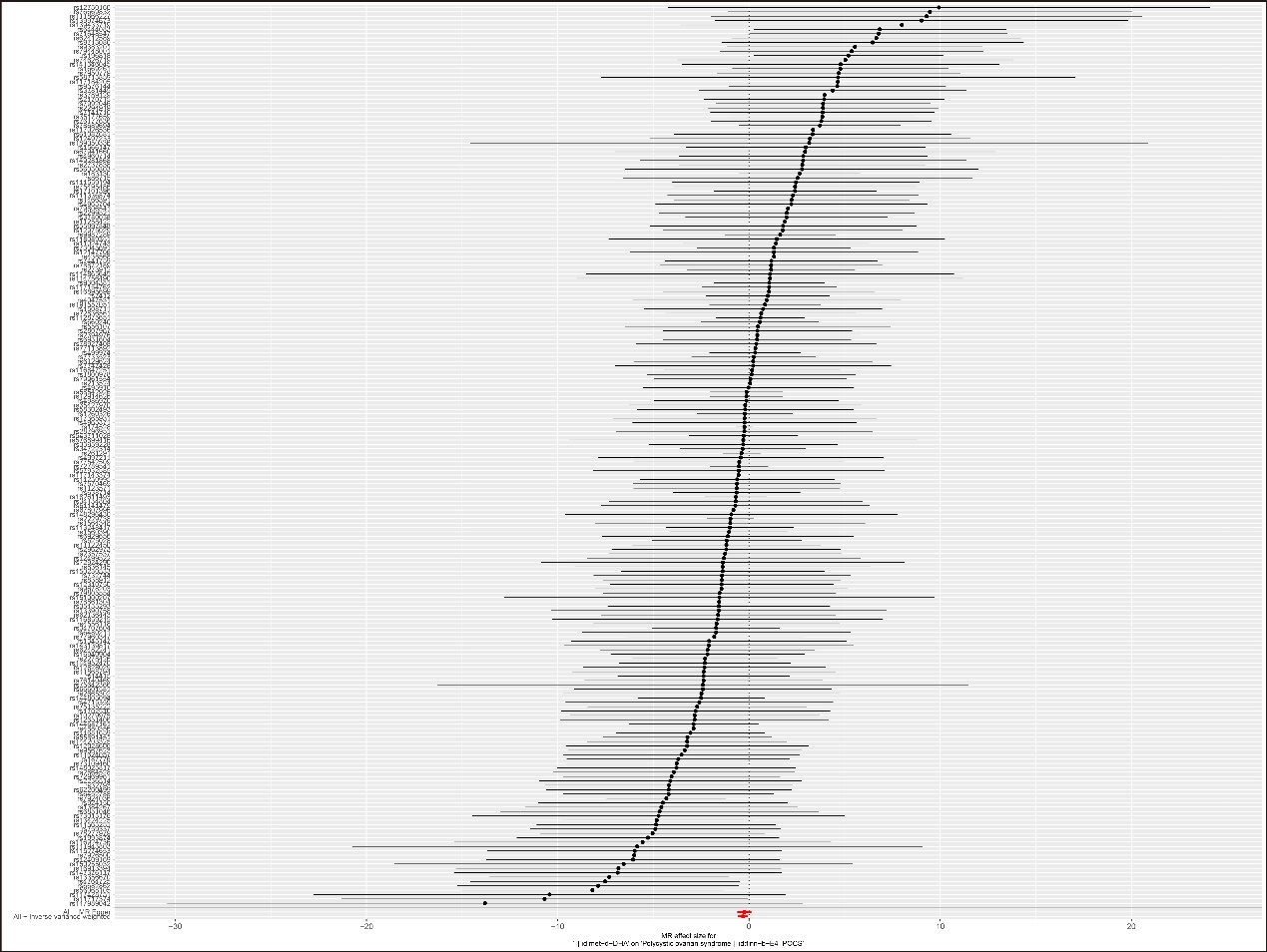


**Supplementary Figure 2. Leave-one-out plot of DHA and PCOS MR Results**


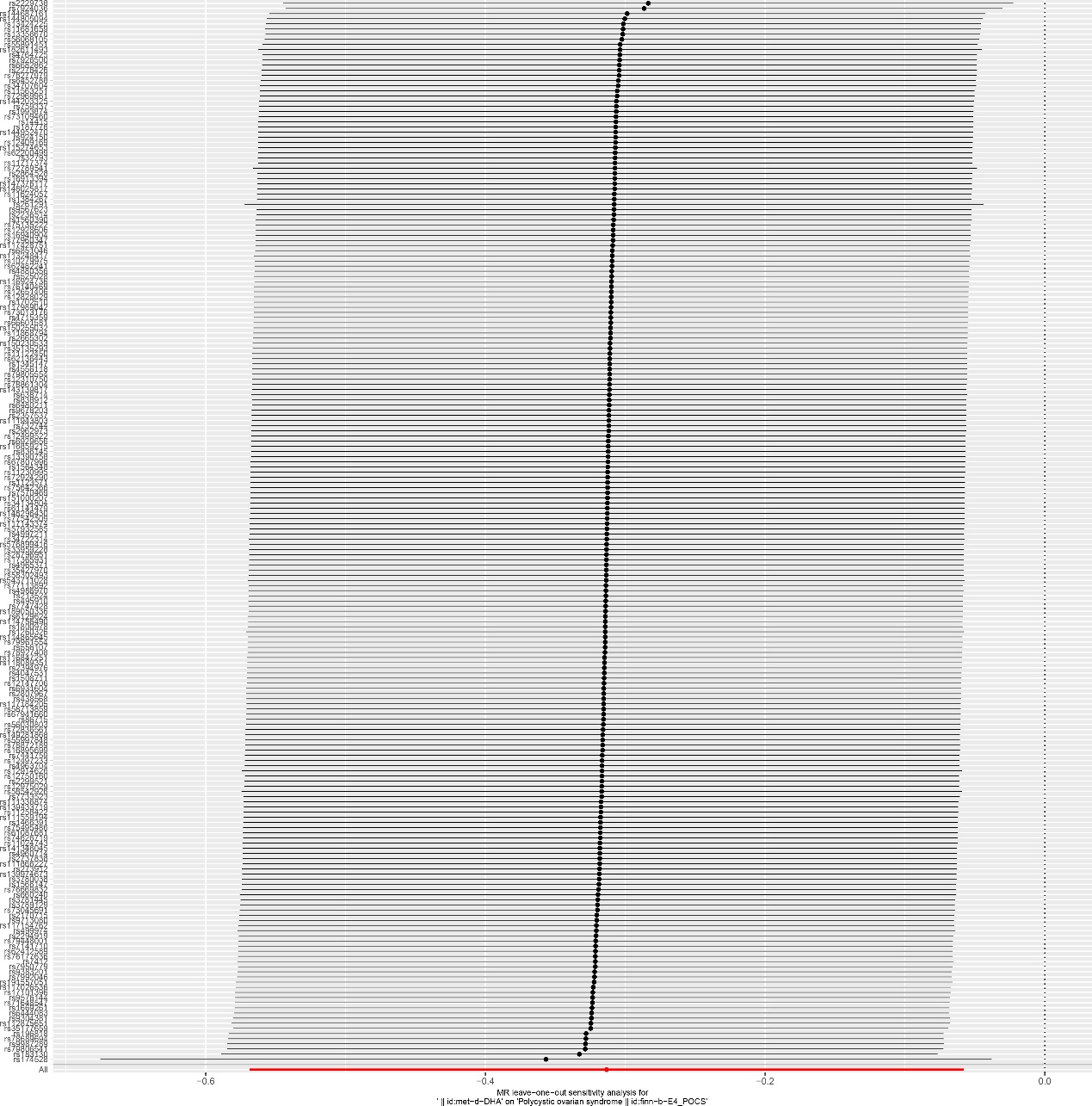

Supplement: Supplementary file 1 [file DataSheet1.docx]
